# Evaluation of a communication skills training to facilitate addressing palliative care related topics in advanced cancer patients: study protocol of a multicenter randomized controlled trial (PALLI-KOM)

**DOI:** 10.1186/s12904-020-00568-3

**Published:** 2020-05-12

**Authors:** Nele Harnischfeger, Hilke M. Rath, Anneke Ullrich, Bernd Alt-Epping, Anne Letsch, Peter Thuss-Patience, Carsten Bokemeyer, Karin Oechsle, Corinna Bergelt

**Affiliations:** 1 Department of Oncology, Hematology and BMT, Palliative Care Unit, University Medical Center Hamburg-Eppendorf, Martinistraße 52, 20246 Hamburg, Germany; 2 Department of Medical Psychology, University Medical Center Hamburg-Eppendorf, Hamburg, Germany; 3 Department of Palliative Medicine, University Medical Center Goettingen, Goettingen, Germany; 4 Department of Hematology, Oncology and Tumor Immunology, Campus Kiel, University Medical Center Schleswig-Holstein, Kiel, Germany; 5 Medical Department, Division of Hematology, Oncology and Tumor Immunology, Campus Virchow Clinic, Charite University Medicine Berlin, Berlin, Germany

**Keywords:** RCT, Palliative care, Education, Communication skills training, Advanced cancer, Oncology, Physician-patient-communication, Early integration of palliative care

## Abstract

**Background:**

Early integration of palliative care concurrently to standard cancer care is associated with several benefits for patients and their caregivers. However, communication barriers on part of the caring physicians often impede a timely referral to palliative care. This study describes the protocol of the evaluation of a communication skills training aiming to strengthen the ability of physicians to address palliative care related topics adequately and early during disease trajectory.

**Methods:**

We will implement a communication skills training and evaluate it within a prospective, multi-centered, two-armed randomized controlled trial (RCT), which will be conducted at four sites in Germany. Eligible subjects are all physicians treating patients with advanced cancer in their daily routine. An intervention group (IG) receiving a group training will be compared to a wait-list control group (CG) receiving the training after completion of data collection. At pre- and post-measurement points, participants will conduct videotaped conversations with standardized simulated patients (SP). Primary outcome will be the external rating of communication skills and consulting competencies addressing palliative care related topics. Secondary outcomes on core concepts of palliative care, basic knowledge, attitudes, confidence and self-efficacy will be assessed by standardized questionnaires and self-developed items. A further external assessment of the quality of physician-patient-interaction will be conducted by the SP. Longitudinal quantitative data will be analyzed using covariate-adjusted linear mixed-models.

**Discussion:**

If the communication skills training proves to be effective, it will provide a feasible intervention to promote an earlier communication of palliative care related topics in the care of advanced cancer patients. This would help to further establish early integration of palliative care as it is recommended by national and international guidelines.

**Trial registration:**

German Clinical Trials Register DRKS00017025 (date of registration: 4 June 2019).

## Background

Complex psychological and physical symptoms of patients with advanced cancer often arise early during disease trajectory [1]. That is why palliative care, which focusses on identifying and addressing various physical, psychosocial and spiritual needs of patients with life-threatening illnesses, should not only be provided at a stadium close to death, but concurrently with oncological treatment [2].

Multiple studies, including several randomized controlled trials, have shown the benefits of early implementation of palliative care concurrently to standard cancer care for patients and their caregivers. It is for example associated with improved symptom control, quality of life and mood, satisfaction with care, coping, understanding of disease and communication about care [3–9]. It can also lead to a decrease in chemotherapy use near end of life and therefore reduce treatment costs [7, 10]. Besides, it has been shown that patients suffering from advanced cancer prefer an early talk about end-of-life topics [11–14].

Addressing palliative care and end-of-life topics should be initiated by the primary care provider of the cancer patient [15, 16]. Since not every patient is cared for by an oncologist, but by other specialists such as gynaecologists, urologists, etc. [17], all physicians treating patients with advanced cancer should aim for early implementation of palliative care into standard oncology care.

Early integration is also recommended by national and international guidelines [2, 15, 18, 19]. The basis and central component of it is communication [20], which at the same time impedes it. Previous findings show that if conversation about palliative care and end-of-life topics occur, then usually too late, when patients are no longer able to decide for their own or already are in crisis [21]. This indicates that physicians seem to avoid referring to palliative care services, which has been confirmed in previous research [22, 23]. Studies and reviews report several reasons and possible barriers to referring to palliative care services. An important barrier is a lack of physicians’ expert knowledge in palliative care, including a wrong understanding of this discipline [24]. A majority of physicians falsely equates palliative care with end-of-life care and associates it with imminent death [16], which often results in the fear of demoralizing the patient when addressing palliative care [23]. Another key reason represents the perceived difficulty to address end-of-life topics [11]. It is reported to be one of the most stressful and difficult parts within oncological treatment [25]. That discomfort is especially based on a high communicative uncertainty [11, 24]. Besides the concern of causing stress in the patients [24], also personal reasons like prior traumatic experiences [22] may lead to a lack of discussing palliative care. Thus, also the physicians’ own attitudes and fears towards death seem to play a role when avoiding such consultations [26, 27].

These aspects illustrate that physicians would benefit from offers to facilitate addressing palliative care in medical consultations with cancer patients. This is confirmed by the fact that physicians themselves demand for more education on communication in palliative care [28]. Strengthening physicians’ communication skills and reducing individual insecurities would as a result promote the timely involvement of palliative care into standard cancer care, as it is required by clinical guidelines [2, 15, 18, 19].

Various studies provide evidence for the effectiveness of communication skills trainings in oncological settings [29–32]. Also, trainings focusing on communication with regard to specific facets of palliative care, such as the transition to palliative care, have shown to be useful [30, 33]. Those trainings in most cases refer to rehearsing one specific conversation, in which the respective topic is communicated [30, 33, 34].

Until now there are no training programs which address the general ability of physicians to talk about different topics relevant to palliative care, to allow for a gradual integration of this field. Also, most existing training programs are designed for oncologists and not open to physicians of different specialisation. Moreover, previous trainings are often very intensive and time-consuming, sometimes lasting multiple days [35].

The objective of this study is to carry out an evaluation of a newly developed communication skills training within a randomized controlled trial. The training aims at strengthening the physicians’ ability to address palliative care related topics adequately and early during disease trajectory.

The main research question is:

- Does the training improve communication skills and consulting competencies addressing palliative care related topics?

Further research questions concerning the effectiveness and acceptance of the training are:

- Does the training improve the quality of physician-patient interaction in conversations about palliative care?
- Does the training support physicians to consider palliative care principles, such as psychosocial needs, within the consultation?
- Does the training improve the perceived confidence of physicians dealing with palliative care related topics?
- Does the training enhance self-efficacy regarding conversations about palliative care?
- Does the training change the physicians’ attitude towards caring for terminally ill patients and communicating about dying and death?
- Does the training improve basic knowledge about palliative care services?
- What is the level of acceptance and satisfaction with the training?

## Methods

### Design

The planned evaluation is designed as a prospective, multi-centered, two-armed randomized controlled trial (RCT).

The aim of the study is to investigate the effect of an intervention with regard to communication about palliative care. It will be carried out at four locations in Germany. Physicians of different specialist qualifications will be recruited and randomly assigned to an intervention group (IG) receiving the communication skills training and a wait list control group (CG). Data of both groups will be assessed 6 to 10 weeks before (baseline, T0) and 6 to 10 weeks after the training (T1). The CG will be assessed at parallel time-points and will attend the training after the end of data collection.

### Cooperation partners

The study will be carried out at four sites with partners with long-term experience in palliative care. Study sites are located at the University Medical Center Hamburg-Eppendorf, the University Medical Center Goettingen, the University Medical Center Schleswig-Holstein and the Charite University Medicine Berlin. Trainings and data collection will be carried out at all four locations. Coordination, training development and data analysis will mainly be conducted in Hamburg, where the principal investigator and the study management are situated.

### Inclusion and exclusion criteria

Eligible for the RCT are physicians who treat cancer patients in their daily routine but are not specialized in palliative care. Participation is not limited to medical oncologists, but open to all physicians treating cancer patients (such as gynecologists, dermatologists, urologists etc.). Participants must provide informed consent for participation in the study. Exclusion criteria are a palliative care specialist training level as well as insufficient German language skills, since the training is conducted in German.

### Development of the intervention

The communication skills training was developed based on literature and four focus groups (with *N* = 28 participants). Within the focus groups the perceptions and needs of physicians treating advanced cancer patients were assessed. Results were merged with relevant aspects from literature, such as the *core competencies in palliative care* defined by the *European Association for Palliative Care* (EAPC) [36]. The training was manualized following the *CReDECI2-Guidelines* (Criteria for Reporting the Development and Evaluation of Complex Intervention in healthcare: revised guideline) [37].

The newly developed communication skills training consists of two sessions each lasting 90 min. It includes short theoretical presentations, written information, audiovisual contents as well as behavioral exercises for active learning. Video material of an expert leading a conversation with a standardized simulated patient (SP) will be presented as a positive example within the training. The intervention is intended to promote the three components of the ‘KSA-Framework’ (Knowledge, Skills, Attitude) [38]. The component ‘knowledge’ includes theory on communication (e.g. specific skills, empathy, dealing with palliative care and end-of-life issues), core principles and concepts of palliative care (e.g. double awareness) and palliative care services. ‘Skills’ will be promoted through rehearsing conversations in role plays with SPs based on case vignettes of advanced cancer patients, which is an effective training strategies for communication skills trainings [39]. ‘Attitude’ will be addressed through the transmission of a new attitude towards palliative care and dealing with advanced cancer patients.

#### Train-the-trainer workshop

As recommended in guidelines on communication skills trainings [32], the study partners who will conduct the training will attend a train-the-trainer workshop, which will be carried out by the coordinating center. The main content will be the correct implementation of the training manual. Moreover, participants will be taught how to train future study staff as well as instructing SPs for the consultations pre and post training in the IG and CG. When SPs will be briefed in the respective study sites, a member from the principal investigator’s team will be present to ensure standardization.

### Evaluation of the training (RCT)

**Fig. 1:**
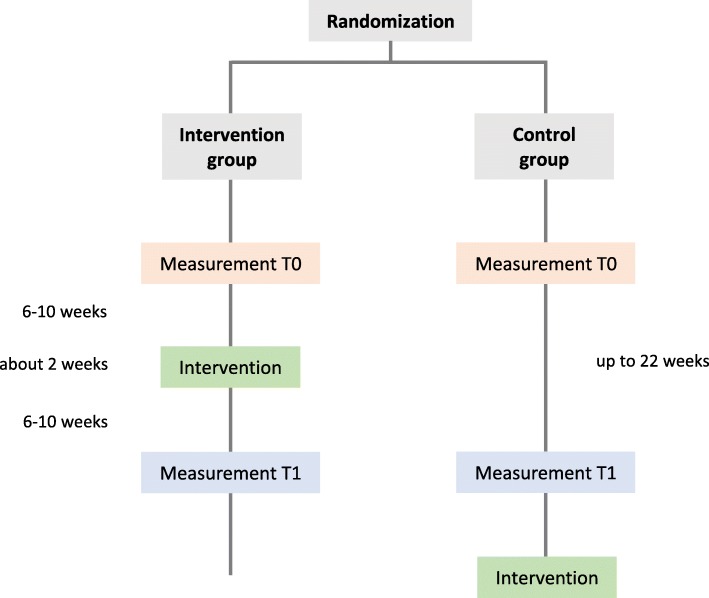
Flowchart of the randomized controlled trial (RCT)

The communication skills training will be implemented and evaluated. The procedure of the RCT is presented in Fig. 1. The IG will receive the training between the two measurement points, while the control group receives it after the end of data collection.

#### Sample size and power

Sample size calculation indicates that a total sample size of 134 participating physicians (67 per group) is needed to detect medium group differences (Cohen’s *d* = 0.5) in a two tailed test with a power of 0.80 (80%) at a significance level of *p* < 0.05. A medium correlation (*r* = 0.3 [40]) between initial level and outcome measurement is assumed. Based on an estimated dropout-rate of 30%, recruitment of 174 physicians (87 per group) is necessary.

This amount of participating physicians will be recruited consecutively in groups of 10 and equally divided between the four study sites. For the recruitment, information material will be spread in local oncological networks, at conferences and events.

#### Randomization

Computer generated randomization for all study sites will be performed by an experienced independent co-worker from the statistical-methods-group of the Department of Medical Psychology at the University Medical Center Hamburg-Eppendorf. Randomization will be conducted separately per site. Since participants will be recruited consecutively, block randomization with block sizes of 20 will be applied. Within every block of 20 participants at each study site, 10 will be assigned to IG and 10 to CG. Allocation ratio of 1:1 will be used.

#### Study intervention

The communication skills training will be conducted with up to 10 physicians per group. Each cooperating site will consecutively run 2–3 training cycles. The training of each group will consist of two sessions, each lasting 90 min. There will be approximately two weeks between both sessions. The training will be carried out by two trainers: the leading investigator of the respective study site with expert knowledge in palliative care and a researcher with expert knowledge in communication and psychooncology. Contents and schedule are standardized through the manual. To ensure adherence, the trainers will fill out a training protocol after the training.

#### Evaluation

The evaluation of the training will be carried out by comparing the pre (T0)-post (T1) differences of consultation performance of the IG and the CG. Therefore, the participants of IG and CG will conduct a videotaped medical consultation on palliative care related topics with a SP at T0 and T1. The consultations will be arranged individually between the physician and the SP and will take place in the familiar working environment of the physician. No further study assistant will be present, so that the setting is kept as naturalistic as possible.

### Outcomes and measurement

Table 1 displays an overview of collected data, instruments, measurement points and sources of data. Measurements consist of external and self-assessed items. The following instruments will be used regarding our primary and secondary study outcomes.

**Table 1:** Overview of study measures

Measures	Instrument	Baseline/ Pre-Training (T0)	Post-Training (T1)	Source of data
Communication skills and consulting competencies addressing palliative care related topics	German version of the COM-ON-Checklist	**●**	**●**	External assessment by researchers
Consideration of palliative care principles	Self-developed questionnaire based on EAPC-competencies	**●**	**●**	External assessment by researchers
Quality of physician-patient-interaction	German version of the Questionnaire on the Quality of Physician-Patient-Interaction (QQPPI)	**●**	**●**	External assessment by simulated patients
Sociodemographic data	Self-developed items	**●**		Physician self-report
Confidence in dealing with palliative care related topics	Self-developed items	**●**	**●**	Physician self-report
Self-efficacy regarding conversations about palliative care	German version of Self-Efficacy in Palliative Care Scale (SEPC)	**●**	**●**	Physician self-report
Attitude towards caring for terminally ill patients	German version of Thanatophobia-Scale	**●**	**●**	Physician self-report
Knowledge about palliative care services	Self-developed items	**●**	**●**	Physician self-report
Acceptance of and satisfaction with training	Self-developed items		**●**	Physician self-report

#### Primary outcome

##### Communication skills and consulting competencies addressing palliative care related topics

In order to assess the consultation performance of the physicians, we will develop different case vignettes, which will be varied systematically between the measurement points. While the severity of the disease will be comparable, the type of symptoms and the patient’s needs will differ from case to case.

Communication skills and consulting competencies addressing palliative care related topics will be assessed by the research team using the German version of the COM-ON-checklist (COM-ON = *Communication in Oncology*) [41], which is a valid instrument for the external assessment of communication behavior of oncologists consisting of 5 scales and 31 Items. Researchers will rate the videotaped physician-patient-consultations on a 5-point Likert scale by the following scales: *general communication skills*, *specific skills: transition to palliative care*, *involvement of relatives* and *overall evaluation items*. The fifth scale named *disclosing information about clinical trials* will be excluded, because in this context no such information has to be given, so that only 21 items are used. The instrument has proved to be valid and reliable [41].

To ensure independent and blinded rating of the consultation, we will rotate the videotapes between the four research sites. Thus, the rater will not have any information on group membership (IG or CG) of the respective participant.

#### Secondary outcomes: external assessments

##### Consideration of palliative care principles

We will develop a study-specific questionnaire consisting of items based on the EAPC-competencies [36] to assess the application of palliative care principles. With this questionnaire the consultation competence regarding palliative care will be externally assessed by the researchers.

##### Quality of physician-patient-interaction

A further external evaluation of the consultation is carried out by the SPs with the German version of the Questionnaire on the Quality of Physician-Patient-Interaction (QQPPI) [42]. A 5-point Likert-scale from (1) strongly disagree to (5) strongly agree is used. The total score is determined by the mean value of all items. The instrument has proved to be valid and reliable [42].

#### Secondary outcomes: self-report

##### Confidence in dealing with palliative care related topics

The physician’s perceived confidence will be assessed by study-specific, self-developed items.

##### Self-efficacy regarding conversations about palliative care

A German version of the Self-Efficacy in Palliative Care Scale (SEPC) [43] will be used to assess self-efficacy. It consists of 23 items, which assess the constructs of self-efficacy and outcome expectancies in palliative care. Each subscale represents different target behaviors and skills, upon which the study participant rates his or her confidence in his or her ability to perform the respective behavior or skill on a 100 mm visual analogue scale ranging from (0) very anxious to (10) very confident. The SEPC is a valid and reliable questionnaire [43].

##### Attitude towards caring for terminally ill patients

To measure this construct, we will make use of the Thanatophobia-Scale [44], a short questionnaire that addresses feelings associated with fear of death among healthcare professionals. The 7 items measure discomfort in dealing with dying patients on a seven-point Likert scale ranging from (1) strongly disagree to (7) strongly agree. A total score is calculated by summing all items. The Thanatophobia-Scale has proved to be valid and reliable [43, 44].

##### Knowledge about palliative care services

Basic knowledge concerning palliative care services will be assessed by study-specific, self-developed items.

##### Acceptance of and satisfaction with the training

The participants’ perception concerning the quality of the training will be assessed after the training by study-specific, self-developed items, supplemented by few open questions.

### Data analyses

This study is a multivariate, two-armed RCT with repeated measurement points across time. Descriptive statistics will be used to characterize and describe groups (IG and CG). Using univariate analyses (t-test, analysis of variance) or comparable non-parametric tests (chi-square-tests, U-tests), differences between subgroups (e.g. age, medical specialization, level of working experience, work setting) will be explored.

In order to measure the success of the training, covariate-adjusted linear mixed models will be conducted for analyzing primary and secondary outcome variables. The considered covariates will be the initial level of outcome variables at T0. This method allows to also consider missing data. For non-repeated continuous and binary measurements, ordinary linear regression and logistic models will be used.

### Study status

The project duration is 36 months. The study was initiated in March 2019. The first 12 months are dedicated to the first study phase including extensive preparatory work, focus groups as well as development and manualization of the training. Recruitment of participants will start in April 2020. Data collection of both measurement points is planned to be completed by October 2021.

### Ethics and consent

We will provide written information material to study participants and SP, who both must provide informed consent before data collection. The study protocol and other requested documents were reviewed and approved by the medical ethics committee of the Medical Chamber of Hamburg (date: 19 November 2018, number: PV5910).

### Data confidentiality

To ensure confidentiality, data will be stored pseudonymized on a secure database in accordance with the General Data Protection Regulations [45]. Information and measurements of the study participants collected during the study will be stored separately from the personal information. Extensive considerations on data protection with the data protection officer of the University Medical Center Hamburg-Eppendorf were carried out.

## Discussion

The aim of this study project is to evaluate a communication skills training that addresses physicians’ ability to communicate adequately and early about palliative care related topics. To the best of our knowledge, this is the first randomized controlled trial for such training. Few communication skills trainings on palliative care related topics do exist, but those solely focus on communicating single, specific facets of palliative care [30, 33, 34].

earlyEarly integration of palliative care into the care of advanced cancer patients is recommended by various guidelines [2, 15, 18]. However, different barriers on part of the physicians impede this. A major barrier and reason for the avoidance of referring to palliative care is communicating the associated topics to patients, which at the same time is regarded a fundamental component of integration [20]. Once completed, the project will provide an evidence-based approach to reduce those communication barriers. Accordingly, future use of the training program will promote an earlier referral to palliative care in the care of advanced cancer patients. This is associated with several advantages not only for patients and their caregivers, but also for the health care system, as it reduces costs [15] by providing less aggressive care at the very end of life [46]. Since the training is designed for physicians of different specialization treating cancer patients, the intervention addresses a broad clientele. The assessment of several outcome variables with external and self-assessment instruments in our study will allow for a comprehensive, multiperspective insight into the effectiveness of the training. This will enable conclusions on future interventions, further research and the feasibility of similar trials. A limitation of the study represents a potential selection bias. Due to the voluntary participation, it might be that those physicians take part, who already have a higher interest or competence in physician-patient communication. However, ethical guidelines prevent a subsequent non-responder analysis, so that this circumstance cannot be analyzed.

The study results will be disseminated through publication in peer-reviewed scientific journals and presentations on scientific meetings and conferences.

In conclusion, this randomized controlled trial will provide a feasible intervention designed for a broad range of recipients to promote earlier communication about palliative care and end-of-life topics.

## Data Availability

Not applicable.

## Abbreviations

CG: Control group
COM-ON: Communication in Oncology
CReDECI2: Criteria for Reporting the Development and Evaluation of Complex Intervention in healthcare
EAPC: European Association for Palliative Care
IG: Intervention group
KSA: Knowledge, Skills, Attitude
QQPPI: Questionnaire on the Quality of Physician-Patient-Interaction
RCT: Randomized controlled trial
SEPC: Self-Efficacy in Palliative Care Scale
SP: Simulated patients
